# Evaluation of Selected Biometric Parameters in Cataract Patients—A Comparison between Argos^®^ and IOLMaster 700^®^: Two Swept-Source Optical Coherence Tomography-Based Biometers

**DOI:** 10.3390/medicina60071057

**Published:** 2024-06-27

**Authors:** Mateusz Porwolik, Agnieszka Porwolik, Ewa Mrukwa-Kominek

**Affiliations:** 1 Department of Ophthalmology, Faculty of Medical Sciences in Katowice, Medical University of Silesia, 40-055 Katowice, Poland; emrowka@poczta.onet.pl; 2 Department of Ophthalmology, Kornel Gibiński University Clinical Center, Medical University of Silesia, 40-055 Katowice, Poland; 3 Silesia Orthodontics, Juliusza Słowackiego 13, 40-094 Katowice, Poland; pekalaa94@gmail.com

**Keywords:** individual refractive index, equivalent refractive index, eye biometry, IOL calculation, swept-source optical coherence tomography, SS-OCT, cataract surgery, Argos^®^, IOLMaster 700^®^, enhanced retinal visualization

## Abstract

*Background and Objectives*: To compare the biometry of eyes obtained with two swept-source optical coherence tomography-based biometers—Argos (A), using an individual refractive index, and IOLMaster 700 (IM), using an equivalent refractive index—for all structures. *Materials and Methods*: The biometry of 105 eyes of 105 patients before cataracts were analyzed in this study. Parameters such as axial length (AL), anterior chamber depth (ACD), and lens thickness (LT) were compared from both devices. According to the axial length measurements, patients were divided into three groups, as follows: group 1—short eyes (AL < 22.5 mm), group 2—average eyes (22.5 ≤ AL ≤ 26.0 mm), and group 3—long eyes (AL > 26.0 mm). *Results*: The correlation coefficiency among all compared parameters varies from R = 0.92 to R = 1.00, indicating excellent reliability of IM and A. A statistical significance in axial length was indicated in the group of short eyes (n = 26)—mean AL (A) 21.90 mm (±0.59 mm) vs. AL (IM) 21.8 mm ± (0.61 mm) (*p* < 0.001)—and in the group of long eyes (n = 5)—mean AL (A) 27.95 mm (±2.62 mm) vs. mean AL (IM) 28.10 mm (±2.64) (*p* < 0.05). In the group of average eyes (n = 74), outcomes were similar—mean AL (A) 23.56 mm (±0.70 mm) vs. mean AL (IM) 23,56 mm (±0.71 mm) (*p* > 0.05). The anterior chamber depth measurements were higher when obtained with Argos than with IOLMaster 700—mean ACD (A) 3.06 mm (±0.48 mm) vs. mean ACD (IM) 2.92 mm (±0.46) *p* < 0.001. There was no statistical significance in mean LT—mean LT (A) 4.75 mm (±0.46 mm) vs. mean LT (IM) 4.72 mm (±0.44 mm) (*p* = 0.054). The biometry of one eye with dense cataracts could be measured only with Argos, using the Enhanced Retinal Visualization mode. *Conclusions*: Axial length measurements from both devices were different in the groups of short and long eyes, but were comparable in the group of average eyes. The anterior chamber depth values obtained with Argos were higher than the measurements acquired with IOLMaster 700. These differences may be particularly important when selecting IOLs for patients with extreme AL values.

## 1. Introduction

Cataract surgery is a commonly performed ophthalmic procedure that involves replacing the cloudy natural lens in an eye with an artificial intraocular lens. The positive outcome of this surgery greatly depends on thorough preoperative planning, namely adequate estimation IOL power. This calculation is dependent on accurate measurements of several ocular parameters. Those parameters are nowadays acquired using biometers, as a possible patient-friendly and easy-to-use alternative to ultrasound measurements, which have been used for more than 20 years in clinical applications. The biometers’ precision is higher than that of the applanation method, and can be compared only with high-precision immersion ultrasound due to the optimization of IOL’s constants [1].

The technique of optical biometry was introduced into the market in September 1999 by Carl Zeiss by bringing out the IOLMaster in Germany, which was approved by the U.S. Food and Drug Administration (FDA) in March 2000 [2]. Optical biometry measurements using partial coherence interferometry (PCI) produce better IOL power predictions and refractive outcomes following cataract surgery than ultrasound biometry measurements [3,4,5]. Nevertheless, variability in the biometry measurements obtained using different types of biometer models can create some errors within these critical calculations, undermining their effectiveness, as well as leading to inappropriate estimates for IOL power and visual outcomes following cataract surgery. Axial length is the largest source of error in intraocular lens power calculations, with a mean absolute error (MAE) of 0.6 diopter (D) for an eye of average dimensions [6]. According to a survey conducted by Mamalis et al. and Jin et al., incorrect IOL power still remains one of the most common reasons for surgical intervention or IOL exchange [7,8]. According to a meta-analysis comparing the technical failure rate (TFR) of SS-OCT vs. PCI for low-coherence optical reflectometry (LCOR) methods, a significantly decreased TFR was revealed among SS-OCT’s biometry compared to PCI/LCOR devices [9]. In this field, the introduction of swept-source optical coherence tomography (SS-OCT) in 2014 [10] was the next evolutionary step in providing higher precision and reliability by providing images with a lower signal-to-noise ratio (SNR) [11]. SS-OCT is nowadays considered as the golden standard in preoperative axial measurements [12]. SS-OCT calculates the AL of the eye by using the segmental or equivalent refractive indices of each measured structure [13]. Computationally, the AL data for each segment calculated with the segmental refractive indices for each segment is more accurate than the data calculated using the single equivalent refractive index for the entire eye; it is most suitable for nonstandard anatomical eyes since the ratio of each part is different. For instance, the AL of a long eye includes the anatomically average magnitude of anterior segment length and long vitreous cavity length; therefore, an examination using the single equivalent refractive index of the eye seems to produce a short AL because of the lower refractive index of the vitreous body compared to the equivalent index [14]. This article presents a comparative analysis of eyes’ biometry obtained with two swept-source optical coherence tomography-based biometers—Argos^®^ (A) (Movu Inc., Komaki, Japan) and IOLMaster 700^®^ (IM) (Carl Zeiss Meditec, Jena, Germany). 

## 2. Materials and Methods

### 2.1. Study Population

This study was conducted at the Department of Ophthalmology, Professor K. Gibinski University Clinical Center, Medical University of Silesia, Katowice, Poland. Informed consent was obtained from all patients before beginning data collection and analyses. The biometry of 105 eyes of 105 patients before cataract surgery with the implantation of an intraocular lens were analyzed in this study. Only one eye of each patient was included in the study; if one eye had already undergone previous cataract surgery, the other eye was included. If neither eye had previously undergone cataract removal surgery, the eye was randomly selected for analysis. All the measurements were conducted under non-dilated pupil conditions with the artificial ambient clinic light. In all the measurements, the patients were positioned into a comfortable state with the help of the forehead and chin rests of each biometer. The exclusion criteria were as follows: previous corneal or intraocular surgery, missing data, corneal disease or ocular surface disorders, a history of ocular trauma, and/or severe systemic disease. Ocular examinations including refraction, visual acuity, slit-lamp examination, intraocular pressure measurement, and fundus examination were carried out before the operation.

### 2.2. Comparing Biometers 

Argos uses a 1060 nm wavelength and a 20 nm bandwidth swept-source technology for collecting 2D OCT 3 000 A-scans/s. The measured optical distances are then converted into geometric distances by using the standard refractive indices for different parts of the eye. The refractive indices used here are 1.374 for the cornea, 1.336 for the anterior chamber and vitreous humor, and 1.41 for the lens. 

In principle, it is important to note that the sensitivity of OCT decreases with depth. In practice, in the case of a dense cataract, it is impossible to examine the retinal pigment epithelium layer or measure the vitreous length due to the reduced light energy. Argos is equipped with the Enhanced Retina Visualization (ERV) mode, whereby the length of the optical path is determined through minimizing the influence of attenuation and by moving the OCT’s sensitive point to the retinal side using the same concept, as in choroidal imaging using the technology of enhanced depth imaging [15].

The IOLMaster 700 is also a swept-source optical coherence tomography like Argos; however, it has a wavelength of 1050 nm and uses a refractive index of 1.3375 for the biometry of all segments [16], with a scanning rate of 2000 scans/s, a 44 mm scan depth, and a 22 μm resolution in tissue to obtain a 2D OCT scan of the eye [10]. There is no dedicated mode for advanced cataract measurements. 

### 2.3. Comparing Results 

Biometry parameters from both devices, such as axial length (AL), anterior chamber depth (ACD), and lens thickness (LT), were compared. As IM was considered as the reference biometer and the A was considered as the new biometer, data were grouped according to the IM measurements. According to the axial length measurements, patients were divided into three groups, as follows: group 1—short eyes (AL < 22.5 mm), group 2—average eyes (22.5 ≤ AL ≤ 26.0 mm), and group 3—long eyes (AL > 26.0 mm).

### 2.4. Statistical Analysis

Agreement between the studied measurements (AL, ACD, and LT) was analyzed using the Bland–Altman method, with the determination of the average offset between the results and the concordance intervals with their 95% confidence intervals. *p* values less than 0.05 were considered statistically significant [17]. Based on the 95% confidence interval of the intraclass correlation coefficient (ICC) estimate, values of less than 0.5, of between 0.5 and 0.75, of between 0.75 and 0.9, and of greater than 0.90 are indicative of poor, moderate, good, and excellent reliability [18]. The Pearson correlation coefficient (R) was used to evaluate the inter-device correlation. All results were visualized with graphs. Data were analyzed using Statistica 13.3 software, StatSoft (Hamburg, Germany).

### 2.5. Ethics Approval and Informed Consent

Written informed consent was obtained from all patients, and the study protocol was approved by the institutional committee on human research, ensuring that it conformed to the ethical guidelines of the 1975 Declaration of Helsinki.

## 3. Results

### Correlation Analysis

**Figure 1:**
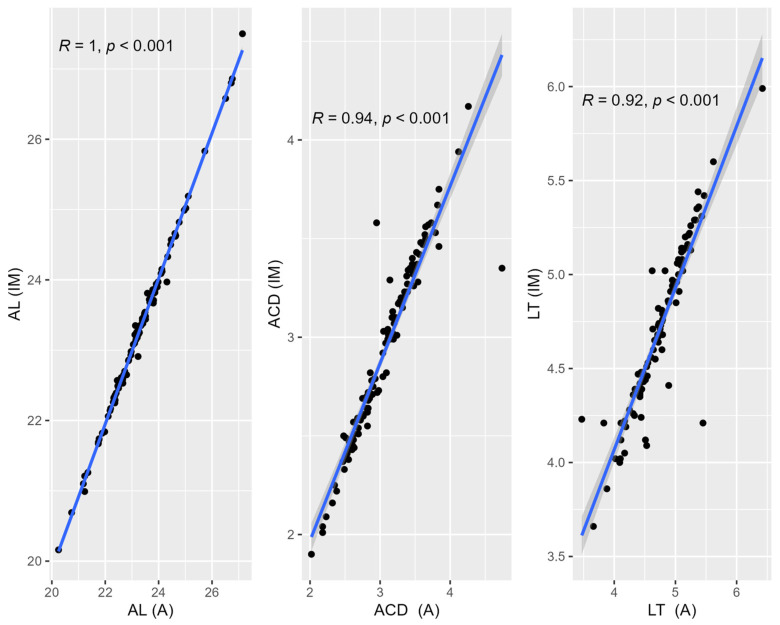
Correlation analysis and determination of regression lines for axial length (AL), anterior chamber depth (ACD), and lens thickness (LT).

A correlation analysis and the determination of regression lines for the following measurements were obtained: axial length (AL), anterior chamber depth (ACD), lens thickness (LT); the correlation coefficients ranged from 0.92 to 1.00, indicating the excellent reliability of IM and A (Figure 1).

In order to determine the agreement between the biometry devices, a Bland–Altman analysis was carried out. As a preliminary analysis showed no difference between the IOLMaster and Argos, all the data were used in the agreement analysis.

**Figure 2:**
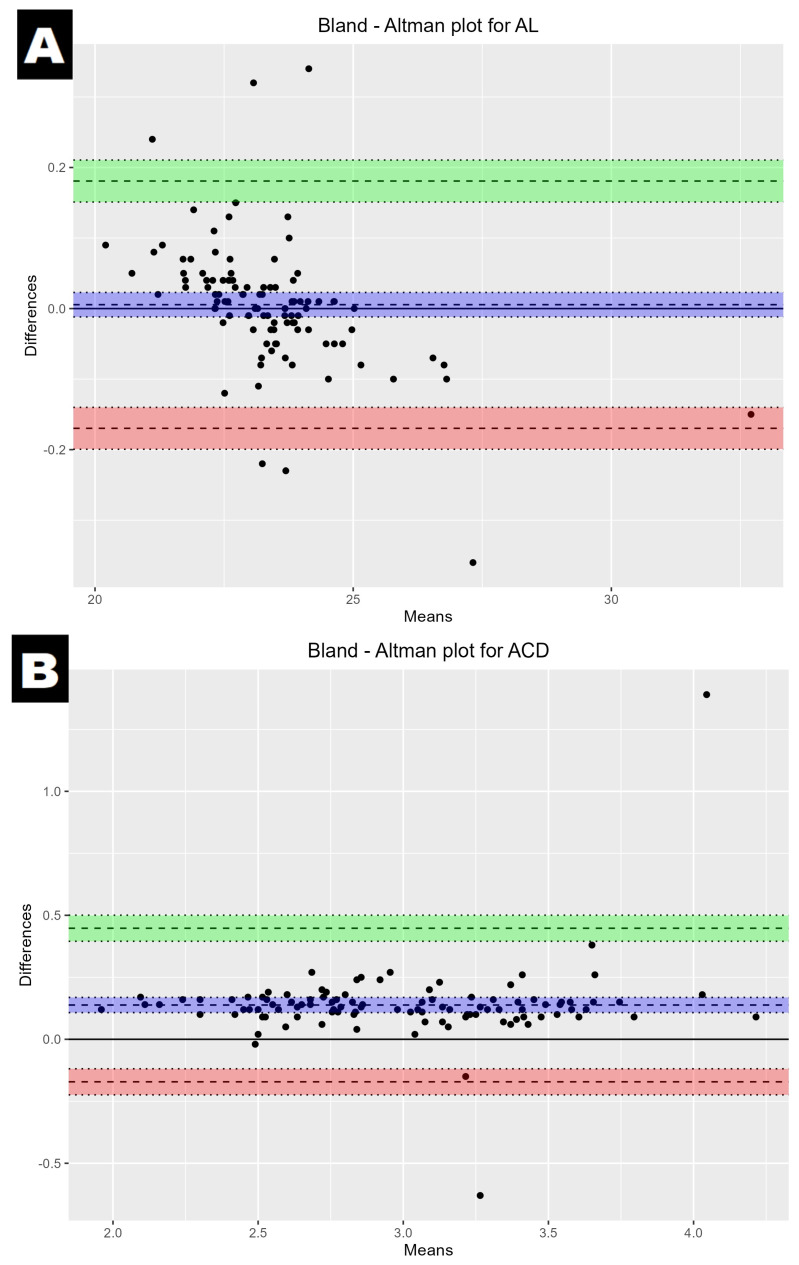

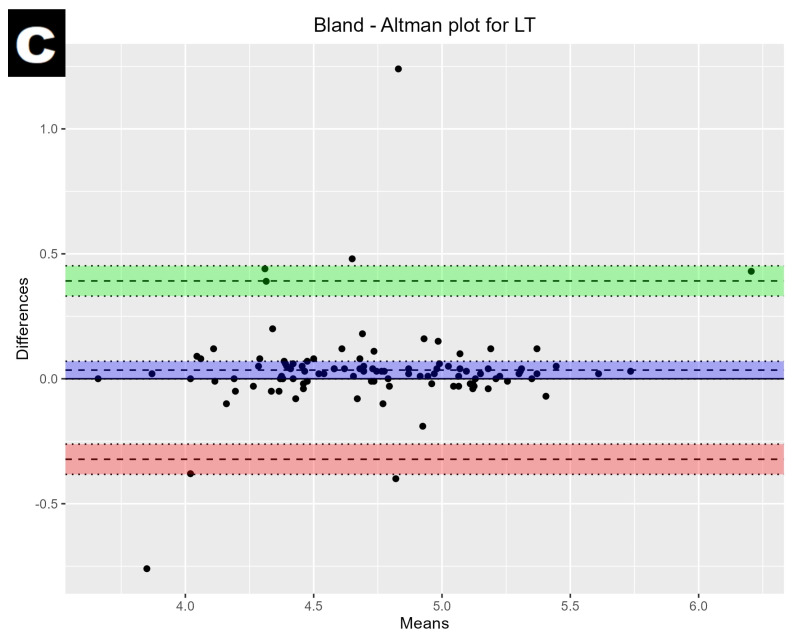
Bland–Altman plot showing the mean difference and LoA between the IOLMaster700 and Argos; axial length (**A**), anterior chamber depth (**B**), and lens thickness (**C**). The mean difference is represented by a solid line, and the 95% LoA is represented by the dashed lines and green/red colours.

The Bland–Altman plot showed a good agreement with a narrow 95% LoA (Table 1 and Figure 2).

**Figure 3:**
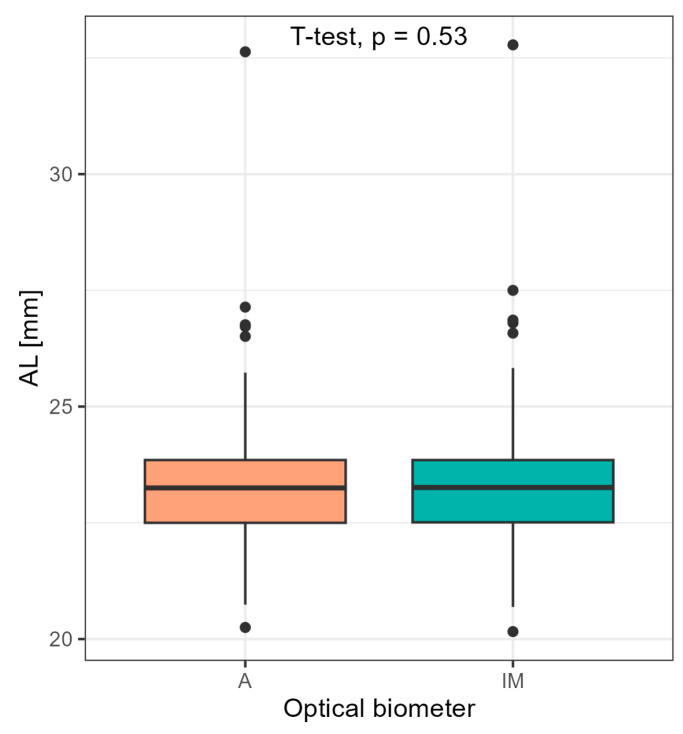
Comparison of axial length for all 105 eyes obtained with Argos and IOLMaster 700 using Student’s *t*-test.

No statistical significance in axial length difference was indicated in the group of all 105 eyes between both devices (*p* = 0.53) (Figure 3). 

**Figure 4:**
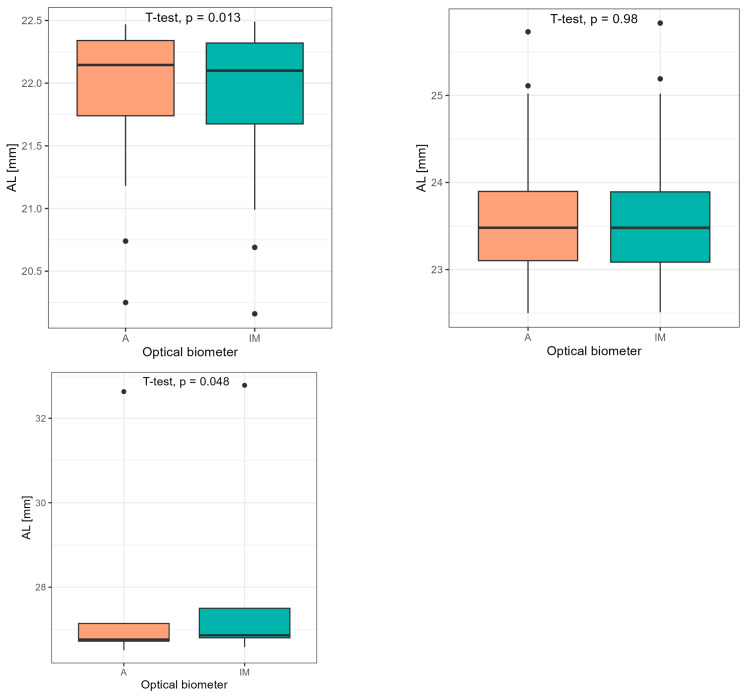
Comparison of axial length values divided on the basis of axial length into the following: group 1—short eyes (AL < 22.5 mm), group 2—average eyes (22.5 ≤ AL ≤ 26.0 mm), and group 3—long eyes (AL > 26.0 mm) using Student’s *t*-test.

More surprisingly, statistically significant differences are revealed when the group of 105 eyes is divided on the basis of axial length into the following: group 1—short eyes (AL < 22.5 mm), group 2—average eyes (22.5 ≤ AL ≤ 26.0 mm), and group 3—long eyes (AL > 26.0 mm). A statistical significance in axial length was indicated in the group of short eyes (n = 26)—mean AL (A) 21.90 mm (±0.59 mm) vs. AL (IM) 21.8 mm ± (0.61 mm) (*p* < 0.001)—and in the group of long eyes (n = 5)—mean AL (A) 27.95 mm (±2.62 mm) vs. mean AL (IM) 28.10 mm (±2.64) (*p* < 0.05). In the group of average eyes (n = 74), outcomes were similar—mean AL (A) 23.56 mm (±0.70 mm) vs. mean AL (IM) 23.56 mm (±0.71 mm) (*p* > 0.05) (Figure 4). 

**Figure 5:**
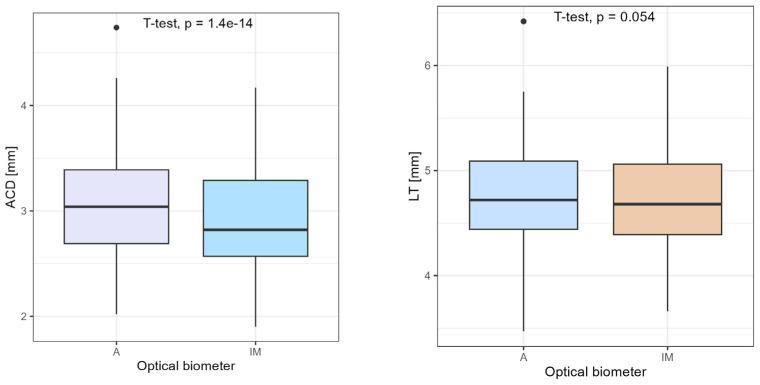
Comparison of ACD and LT using Student’s *t*-test.

Anterior chamber depth measurements were higher when obtained with Argos than with IOLMaster 700—mean ACD (A) 3.06 mm (±0.48 mm) vs. mean ACD (IM) 2.92 mm (±0.46) *p* < 0.001. There was no statistical significance in mean LT—mean LT (A) 4.75 mm (±0.46 mm) vs. mean LT (IM) 4.72 mm (±0.44 mm) (*p* = 0.054), as proven using Student’s *t*-test (Figure 5).

**Figure 6:**
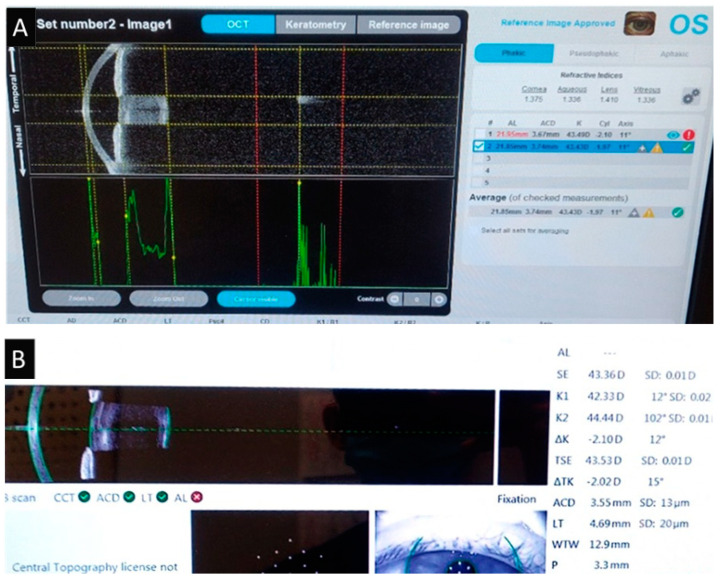
Measurement of dense cataract. Argos’ Enhanced Retinal Visualization mode (**A**) and standard mode measurement of IOLMaster 700 (**B**). * means that the result is statistically significant.

The biometry of one eye with dense cataracts could be measured only with the Argos, using the Enhanced Retinal Visualization mode. The biometry was not possible to measure in this patient using the standard mode on both devices (Figure 6).

## 4. Discussion

According to a summarized review in which twenty-nine studies were discussed, it was revealed that SS-OCT biometers provide excellent repeatable and reproducible outcomes. Despite such results, authors suggest that a range so broad might have an important impact on IOL power calculation when using new-generation formulas [10]. The Bland–Altman 95% limits of agreement indicated excellent agreement between both devices. This finding was in line with previous research, whereby Argos provided good agreement and repeatability compared with the reference biometer [19]. 

No statistical significance in axial length difference was indicated between both devices by comparing all eyes together. However, statistically significant differences between the AL measured using both devices were highlighted when we divided the eyes into three groups. In longer eyes, the proportions of vitreous to AL are bigger, having a relatively larger amount of vitreous in the total AL, whereas shorter eyes fall into a category of a relatively larger proportion of lens thickness in the total AL. In the cases of normal AL ranges, the corresponding refractive index does not affect the results of AL measurement procedures, and our results also do not indicate such marked differences. Chan Min Yang et al. also report similar results to our study; the AL calculated with Argos was shorter in long eyes, with an AL of greater than 26.0 mm (*p* < 0.001), and was longer in the short eyes, with an AXL of less than 22.5 mm (*p* < 0.001, *p* = 0.005) [19]. The other three studies comparing AL measurements from the single refractive index devices were, on average, too short in short eyes and too long in long eyes, when compared to the calculated measurements based on multiple refractive indices. Studies were consistent with our study in that most of the accuracy improvements are noted in short and long eyes [20,21,22]. On the other hand, the mean AL differed significantly (*p* < 0.001) with the IM compared with the A, according to Omoto et al. [13]. A further correction factor to the AL value calculated using conventional biometer devices has also been determined by Wang et al. to account for the offset from the real AL in eyes with ALs > 25 mm before using it in the IOL power formula [23]. The most significant value of this study is that patients with eyes with extreme AL axial values may benefit most from the use of a correlated refractive index during ocular biometry. A further refinement of IOL calculation formulas is key, in the face of increasingly high-tech premium lenses and increasing patient demands.

Our example of patients with dense cataracts is consistent with research showing that 93.4% of dense cataract cases were successfully measured using Argos—with standard or ERV mode [24]. Moreover, the OCT of the SS-OCT module employs a longer central wavelength of 1060 nm, which penetrates further in the cataract tissue than with the PCI and LCOR units, in which wavelengths are directed at 840 nm for the OLCR unit and at 780 nm for the PCI unit. Moreover, the SS-OCT device accurately measured AL in 96% of cases versus 79% for the OLCR device and 77% for the PCI device [25]. Further studies of patients with dense cataracts are needed to better determine the capabilities of the Enhanced Retinal Visualization mode.

The main limitations of this study include the fact that only two devices were compared, as well as the fact that an insufficient number of patients with very dense cataracts were enrolled and that the procedure was performed by different operators, making it difficult to assess postoperative refractive outcomes.

## 5. Conclusions

The IOLMaster 700 and Argos Alcon are both highly advanced swept-source OCT-based biometers capable of measuring with high precision the axial length of an eye. The ocular biometric measurements taken with both devices showed strong agreement. The axial length measurements with both devices were different in the groups of short and long eyes, but were comparable in the group of average eyes. The anterior chamber depth values obtained with Argos were higher than the measurements acquired with the IOLMaster 700. Lens thickness measurements were similar with both devices. Some eyes with dense cataracts could be measured only using Argos. The choice between them should be based on the specific requirements of the practice, including the types of patients commonly seen, the preferred workflow, and budget considerations. Optical biometric technology is advancing, resulting in more accurate outcomes in cataract surgery and IOL implantation. Argos may be a better choice for frequent artificial lens calculations for patients with extreme axial length values as the proportion between each part varies from eyes with standard AL. Through the careful review and statistical comparison of measurements made on both biometers in a cohort of cataract patients, the investigators highlight the possible inconsistencies that may easily go unnoticed. However, such systematic inaccuracies, if not corrected, would inevitably introduce underestimations or overestimations of IOL power, manifesting as compromised refractive results following surgery.

## Figures and Tables

**Table 1:** Bland–Altman LoA for AL, ACD, and LT. Comparison of IOLMaster 700 and Argos.

Parameter	95% LoA (mm)	SEM (Standard Error of the Mean)
AL	−0.170 to 0.181	0.008
ACD	−0.171 to 0.448	0.015
LT	−0.322 to 0.391	0.0178
